# Native *V. californicum* Alkaloid Combinations Induce Differential Inhibition of Sonic Hedgehog Signaling

**DOI:** 10.3390/molecules23092222

**Published:** 2018-09-01

**Authors:** Matthew W. Turner, Roberto Cruz, Jordan Elwell, John French, Jared Mattos, Owen M. McDougal

**Affiliations:** 1 Biomolecular Sciences Graduate Programs, Boise State University, 1910 University Drive, Boise, ID 83725, USA; matthewturner1@u.boisestate.edu; 2 Department of Chemistry and Biochemistry, Boise State University, 1910 University Drive, Boise, ID 83725, USA; rcruzromero@usbr.gov (R.C.); jordanelwell@u.boisestate.edu (J.E.); johnfrench@u.boisestate.edu (J.F.); jaredmattos@gmail.com (J.M.)

**Keywords:** hedgehog signaling, *Veratrum californicum*, cyclopamine, HPLC-MS, Shh-Light II cells

## Abstract

*Veratrum californicum* is a rich source of steroidal alkaloids such as cyclopamine, a known inhibitor of the Hedgehog (Hh) signaling pathway. Here we provide a detailed analysis of the alkaloid composition of *V. californicum* by plant part through quantitative analysis of cyclopamine, veratramine, muldamine and isorubijervine in the leaf, stem and root/rhizome of the plant. To determine whether additional alkaloids in the extracts contribute to Hh signaling inhibition, the concentrations of these four alkaloids present in extracts were replicated using commercially available standards, followed by comparison of extracts to alkaloid standard mixtures for inhibition of Hh signaling using Shh-Light II cells. Alkaloid combinations enhanced Hh signaling pathway antagonism compared to cyclopamine alone, and significant differences were observed in the Hh pathway inhibition between the stem and root/rhizome extracts and their corresponding alkaloid standard mixtures, indicating that additional alkaloids present in these extracts are capable of inhibiting Hh signaling.

## 1. Introduction

The Hedgehog (Hh) signaling pathway plays a vital role in embryonic development [1,2]. In mammals, the Hh signaling pathway consists of the secreted ligands Sonic hedgehog (Shh), Desert hedgehog (Dhh) and Indian hedgehog (Ihh); the transmembrane receptor proteins Patched (Ptch1 and Ptch2), the transmembrane signal transducer Smoothened (Smo), and the Gli transcription factors (Gli1, Gli2, Gli3) [3]. In the absence of Hh ligands, Ptch1 prevents the translocation of Smo to the primary cilia, thereby inhibiting the nuclear localization of Gli and suppressing transcriptional activity. Upon binding of Hh ligands to Ptch1, Smo suppression is abolished and downstream pathway activity proceeds, resulting in nuclear translocation and activation of Gli. Although the Hh ligand proteins all act as morphogens and have similar physiological effects, each Hh ligand performs specialized functions due to the spatial and temporal differences in their expression [4]. The Shh signaling pathway is a major regulator of various processes, including cell differentiation and proliferation, and tissue polarity [2,5]. Inhibition of Shh signaling is widely researched because aberrant Shh signaling is a hallmark of many cancers [6,7,8], including prostate, gallbladder, pancreatic, and basal cell carcinoma [9,10,11,12]. Basal cell carcinoma (BCC) is the most common human cancer and is driven predominantly by the hyperactivation of the Hh pathway [13,14,15]. For this reason, a significant number of BCC patients experience a clinical benefit from vismodegib (Erivedge^®^), a Smo inhibitor approved by the US Food and Drug Administration (FDA) to treat metastatic or reoccurring BCC [16]. In phase 2 trials in BCC patients, a majority experienced clinical benefit with vismodegib treatment that included 30% of metastatic BCC patients demonstrating a 30% decrease in visible tumor dimension, and 64% experiencing stable tumor size. In patients with locally advanced BCC, 43% showed a 30% decrease in visible tumor dimension, and 38% demonstrating stable tumor size. However, development of resistance to vismodegib in up to 20% of advanced BCC patients within one year of treatment represents a significant limitation [15,17]. Various studies have implicated amino acid mutations in the vismodegib binding-site in Smo as a mechanism underlying acquired resistance [15,18,19]. Due to adverse side effects and the potential for acquired resistance to vismodegib there is a continued need to investigate novel compounds that target the Hh signaling pathway, and identification of natural products that act as Hh signaling inhibitors continues to be investigated [20,21,22,23].

*Veratrum californicum* (*V. californicum*) is native to the western United States and is rich in steroidal alkaloids, including cyclopamine, veratramine, isorubijervine and muldamine [6,24]. Of these alkaloids, the most notorious is cyclopamine, a teratogen antagonist of the Shh signaling pathway [25]. Interest in *V. californicum* arose in the 1950s when unsettling high incidences of craniofacial birth defects in lambs were observed by shepherds in Idaho. Numerous review articles have recounted the history of scientific interest in *V. californicum*, the efforts undertaken by researchers at the Poisonous Plant Research Laboratory in Logan, UT to identify and validate the causative agents of the observed birth defects, and the chronological order of the isolation and structural elucidation of individual steroidal alkaloids [6,26,27]. However, few reports in the literature have used modern, highly sensitive analytical techniques to examine the full array of steroidal alkaloids in *V. californicum* [28]. Our lab has implemented extraction techniques of the root and rhizome of *V. californicum* aimed at isolating these steroidal alkaloids and characterizing their bioactivity towards Hh signaling using Shh-Light II cell assays [28,29]. In the current study, we used ethanol extraction of the leaves, stems and roots of *V. californicum* to determine if alkaloid ratios in the extract yield synergistic amplification of Hh signaling suppression as compared to traditional single alkaloid activity. The extracts were characterized using liquid chromatography and high resolution electrospray ionization time of flight tandem mass spectrometry, and their biological activity was tested using Shh-Light II cells. The concentrations of cyclopamine, veratramine, isorubijervine and muldamine were determined, and mixtures of commercially available standards were prepared in the same ratios as found in the extracts derived from the leaf, stem and root/rhizome of *V. californicum*. We sought to test whether well-characterized steroidal alkaloids, at ratios consistent with native plant content, exhibited a synergistic effect to inhibit Hh pathway signaling commensurate with plant extract. Additionally, we sought to determine if additional alkaloids present in the *V. californicum* contribute to Hh signaling inhibition. Earlier investigations of *V. californicum* alkaloids may have failed to identify less abundant alkaloids that are biologically significant and potentially valuable novel Hh pathway signaling antagonists. 

## 2. Results

### 2.1. Qualitative Comparison of V. californicum Alkaloids by Plant Part

**Figure 1:**
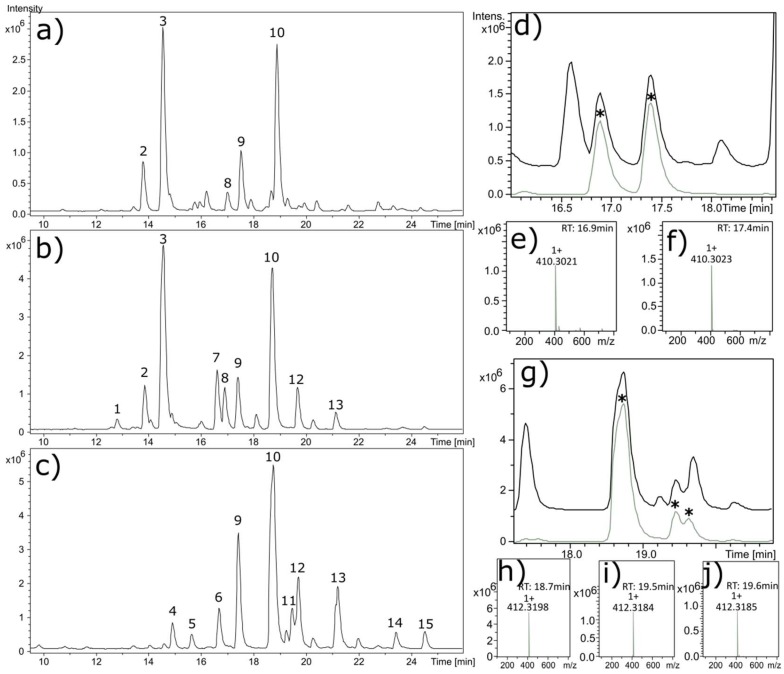
Alkaloid chromatograms for biomass extracts from the (**a**) leaf, (**b**) stem, and (**c**) root/rhizome of *V. californicum*. Common and unique alkaloids identified by MS are observed in each extract. Labelled peaks correspond to the data summarized in Supplemental Table 1. Additional observed peaks that are not labelled did not have molecular formulas consistent with jervine-type alkaloids. Extracted ion chromatograms (EIC) are shown in (**d**,**g**) demonstrating the presence of veratramine and cyclopamine isomers in stem and root/rhizome extracts, respectively. The total ion chromatogram is shown in (**d**) for the stem extract (black) and EIC (grey) generated using the *m*/*z* window 410.3023 ± 0.01. The mass spectra for the peaks indicated by * in (**d**) are shown in (**e**,**f**). The total ion chromatogram is shown in (**g**) for the root extract (black) and the EIC (grey) generated *m*/*z* window 412.3186 ± 0.02. The mass spectra for the peaks indicated by * in (**g**) are shown in (**h**–**j**).

Qualitative variation is observed in the alkaloid composition of *V. californicum* by plant part. The alkaloid profiles of the extracts from the leaf, stem and root/rhizome of *V. californicum* are shown in Figure 1a–c. Identification of each alkaloid peak was achieved by high resolution mass spectrometry and verified by elution time compared to commercially available standards. Data for the most prominent peaks labelled in Figure 1a–c including retention time, *m*/*z*, molecular formula (MF) and alkaloid identity are summarized in Table 1. Mass spectra showing the *m*/*z* for each alkaloid used to estimate molecular formulas listed in Table 1 are shown in Supplemental Figure S1.

Cyclopamine (Peak 10, *m*/*z* 412.3186) and veratramine (Peak 9, *m*/*z* 410.3023) were observed in each of the three plant part extracts. Alkaloids present in both the stem and leaf extracts include cycloposine (Peak 3, *m*/*z* 574.3699) and veratrosine (Peak 4, *m*/*z* 572.3530), which are glycosylated cyclopamine and veratramine, respectively. Peak 1 is a glycosylated alkaloid observed only in stem extract, with an *m*/*z* of 576.3836, corresponding to molecular formula C_33_H_53_NO_7_. In the stem and root/rhizome extracts, isorubijervine (Peak 12, *m*/*z* 414.3342) and muldamine (Peak 13, *m*/*z* 458.3587) are both observed.

Peaks 4, 5, 6, 14 and 15 in Figure 1c correspond to unique alkaloids present only in the root/rhizome extract. These alkaloids have *m*/*z* of 414.3337, 430.3282, 428.3136, 400.3550 and 456.3446 and correspond to the estimated molecular formulas of C_27_H_43_NO_2_, C_27_H_43_NO_3_, C_27_H_41_NO_3_, C_27_H_45_NO and C_29_H_45_NO_3_, respectively. Potential cyclopamine isomers were observed in the root extract, with a *m*/*z* consistent with cyclopamine observed to elute with three distinct retention times. Figure 1g shows the extracted ion chromatogram (EIC) for cyclopamine generated using the *m*/*z* window 412.3186 ± 0.02, and the corresponding mass spectra are shown in Figure 1h–j. Figure 1d shows the EIC for veratramine using the *m*/*z* window 410.3023 ± 0.01, and the corresponding mass spectra are shown in Figure 1e,f.

### 2.2. Quantitative Analysis of V. californicum Alkaloids 

Quantification of cyclopamine, veratramine, isorubijervine and muldamine in alkaloid extracts were determined using charged aerosol detection and calibration curves generated from commercially available standards, with values of R^2^ greater than 0.99. Extractions were preformed three times, and alkaloid concentrations are shown by plant part in Table 2 as mg of each alkaloid extracted per g of initial biomass ± the standard deviation of the concentration observed in triplicate quantities. The quantity of cyclopamine was determined to be 0.21 ± 0.02 mg/g, 3.23 ± 0.16 mg/g, and 7.38 ± 0.08 mg/g for the leaf, stem and root/rhizome, respectively. 

### 2.3. Bioactivity Evaluation of Combined Standards and Plant Extracts

**Figure 2:**
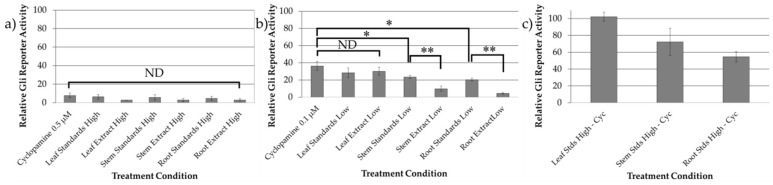
Bioactivity data for cyclopamine alone, the alkaloid standard mixtures, and the plant extracts at (**a**) high concentration (0.5 µM) and (**b**) low concentration (0.1 µM). No significant difference was observed between treatment conditions at high concentration. Statistically noteworthy differences were observed in the low concentration treatments, and * indicates *p* < 0.05, and ** indicates *p* < 0.01. The inhibitory activity of veratramine, isorubijervine and muldamine in the absence of cyclopamine in the same concentrations as the high concentration treatment conditions is shown in (**c**).

Alkaloid standard mixtures were created using commercially available standards in the same ratios as observed in the three plant parts. HPLC was used to validate that the alkaloid standard mixtures matched the concentrations of the ethanolic extract, as is shown for the root/rhizome extract and root standard mixture in Supplemental Figure S2. The bioactivity of these alkaloid standard mixtures were quantified using Shh-Light II cells, and compared to cyclopamine alone at the same concentration, and to *V. californicum* extracts derived from leaf, stem, and root/rhizome of the plant. The treatment conditions evaluating Hh signaling inhibition in Shh-Light II cells are summarized in Supplemental Table S1 with extracts and alkaloid standard mixtures normalized to cyclopamine concentrations of 0.5 and 0.1 µM, referred to as “high concentration” and “low concentration” treatments herein. The results of the biological assays are shown in Figure 2. There is no significant difference between cyclopamine, the alkaloid standard mixtures, and the plant extracts at high concentration treatments shown in Figure 2a. In the low concentration treatments shown in Figure 2b, there is no significant difference observed between cyclopamine standard, and the leaf standard mixture or the leaf extract, indicating that the addition of 0.04 µM veratramine in the standard mixture did not enhance Hh signaling inhibition. No difference is observed between the leaf extract at low concentration and the corresponding combined standard cocktail.

The alkaloid standard mixtures of the stem and root/rhizome samples were significantly different (*p* < 0.05) than cyclopamine alone at the same concentration, with relative Gli-reporter activity determined to 23.56 ± 1.86% and 20.59 ± 1.50% for the stem and root/rhizome, respectively, compared to 36.31 ± 5.13% for 0.1 μM cyclopamine. The inhibitory activity of these compounds was tested in the absence of cyclopamine in the same concentrations as the high concentration treatment conditions, and the results are shown Figure 2c. No Hh inhibition was shown for the leaf standards mixture minus cyclopamine (0.2 μM veratramine), indicating that veratramine does not inhibit the Hh signaling pathway. Modest Hh inhibition was demonstrated for the stem and root/rhizome standard mixtures minus cyclopamine, indicating that isorubijervine and muldamine exhibit Hh antagonism. There is a significant difference (*p* < 0.01) between the stem and root/rhizome extracts and their corresponding alkaloid standard mixtures, signifying that additional alkaloids present in the extracts are capable of inhibiting Hh signaling. 

## 3. Discussion

The current investigation sought to achieve three objectives. The first was to provide a detailed analysis of the alkaloid composition of *V. californicum* based on plant part by performing a quantitative comparison of the alkaloids present in the leaf, stem and root/rhizome of the plant. The second was to evaluate the potential synergistic activity of cyclopamine, veratramine, isorubijervine and muldamine at ratios consistent with alkaloids present in three plant parts, and determine if the alkaloid combinations resulted in more effective Hh pathway antagonism than cyclopamine alone. The third was to determine if additional alkaloids present in the extracts contribute to Hh signaling inhibition by comparing the inhibitory potential of the plant extracts to alkaloid standard mixtures with identical concentrations of cyclopamine, veratramine, isorubijervine and muldamine.

Qualitative differences were observed in the alkaloid composition of *V. californicum* by plant part. Using high resolution mass spectrometry, we identified alkaloids that have previously been unreported for *V. californicum*. The molecular formula and mass of Peak 1 is consistent with that expected for glycosylated isorubijervine or etioline. Glycosylate etioline has previously been reported in the root of *Solanum spirale* [30]. Additional investigation beyond the scope of the current project is required to definitely determine the identify of this alkaloid in *V. californicum*. Etioline is an intermediary in the biosynthetic pathway of cyclopamine, and its presence in the extract may be expected [6]. Peak 4, observed solely in the root/rhizome extract has an *m*/*z* and predicted molecular formula consistent with etioline. In this study, potential cyclopamine isomers (see Figure 1g) were observed in the root/rhizome extract analyzed by LC-MS. One of these potential cyclopamine isomers may be dihydroveratramine, which has previously been identified in *Veratrum album* by Wilson, et al. [31]. However, the relative retention time between dihydroveratramine and cyclopamine, observed by Wilson, et al., does not support this conclusion, because dihydroveratramine (RT: 13.66 min) was observed to elute prior to cyclopamine (RT: 15.09 min), whereas the purported cyclopamine isomer observed in this study elutes after cyclopamine (see Table 1) under similar HPLC conditions. No naturally occurring isomers of cyclopamine have been previously observed in *V. californicum*. 

In Shh-Light II cells using the Dual-Glo^®^ Luciferase Assay System, we evaluated the inhibition of Hh signaling of cyclopamine alone, combinations of alkaloid standards, and the inhibitory potential of extracts from each plant part. As shown in Figure 2a, there is no significant difference between cyclopamine, the alkaloid standard mixtures, and the plant extracts at high concentration treatments. There are trends that indicate enhanced inhibition of alkaloid standard mixtures and extracts compared to cyclopamine alone, but these do not amount to statistically significant differences. The reason for this result may be due to low levels of Gli reporter activity observed in each treatment. However, as demonstrated in Figure 2b, it was determined that addition of 0.1 µM muldamine, veratramine and isorubijervine enhance Hh signaling inhibition significantly when compared to cyclopamine alone, as demonstrated by the stem and root/rhizome standard mixtures compared to cyclopamine alone. Addition of veratramine to cyclopamine does not enhance Hh signaling inhibition as demonstrated by the leaf standard mixture compared to solely cyclopamine. By replicating concentrations of cyclopamine, veratramine, isorubijervine and muldamine observed in plant extracts using commercially available standards and comparing the inhibitory potential of the plant extracts to alkaloid standard mixtures, we determined that additional alkaloids present in the crude stem and root/rhizome extracts inhibit Hh signaling. The alkaloids present in the leaf extract include cycloposine, veratrosine, cyclopamine, veratramine, and the potential veratramine isomer labeled Peak 8 in Figure 1. No difference is observed between the leaf extract at low concentration and the corresponding combined standard mixture. This result indicates that cycloposine, veratrosine and Peak 8 do not contribute to Hh signaling inhibition in this model system. However, it has been proposed that hydrolysis of the glycosidic linkage in glycosylated alkaloids during digestion contributed to the teratogenic effects of *V. californicum* alkaloids when consumed by foraging sheep [32]. Furthermore, no significant difference is observed between cyclopamine alone, the leaf standard mixture or the leaf extract, indicating that the addition of 0.04 µM veratramine in the standard mixture, or the additional alkaloids present in the leaf extract did not enhance Hh signaling inhibition. We observed a significant difference between the alkaloid standard mixtures of the stem and root/rhizome samples and cyclopamine alone. This indicates the addition of veratramine, isorubijervine and muldamine enhance Hh inhibition. However, the modest enhancement of Hh inhibition seems to be additive rather than synergistic, with the addition of these alkaloids providing additional, albeit more weakly inhibitory compounds. No Hh inhibition was demonstrated for 0.2 μM veratramine in the leaf standard minus cyclopamine treatment, indicating veratramine does not inhibit the Hh signaling pathway. This corroborates feeding trials in which veratramine was shown to cause teratogenic malformations in sheep distinct from cyclopia, such as hypermobility of the knee joints leading to bow-legged lambs unable to stand [33]. The stem and root/rhizome standard mixtures containing veratramine, isorubijervine and muldamine indicate that muldamine and/or isorubijervine inhibit the Hh pathway. Muldamine has been shown to result in craniofacial defects in hamsters in feeding studies that may be attributed to interruption of normal Hh signaling [34]. Further investigation to isolate, characterize, and assess the bioactivity of individual, less abundant alkaloids present in the stem and root/rhizome extracts is underway. 

## 4. Materials and Methods 

### 4.1. Chemicals and Solvents

Cyclopamine (>99% purity) was purchased from Alfa Aesar (Ward Hill, MA, USA), veratramine (>98% purity) was purchased from Abcam Biotechnology Company (Cambridge, UK), and isorubijervine (99% purity) and muldamine (99% purity) were purchased from Logan Natural Products (Plano, TX, USA). The purity of the reference standards was verified in house by HPLC-MS. Extraction and purification solvents, 95% ethanol, ammonium hydroxide and chloroform were purchased from Fisher Scientific (Pittsburgh, PA, USA). High pressure liquid chromatography (HPLC) mobile phases included 0.1% formic acid and HPLC grade acetonitrile (>99% purity, Fisher Scientific). 

### 4.2. Sample Extraction and Preparation

A complete specimen of *V. californicum* was harvested in the Boise National Forest, Idaho at an elevation of 2134 m. The leaf, stem and roots/rhizomes of the plant were separated, and all plant parts were cut into smaller pieces to fit into quart size sealable bags. The specimens were placed in a cooler on a bed of ice for transportation. The biomass was collected at a late stage in the plant’s life cycle; the plant had noticeable brown edges along its leaves and top indicating annual deterioration of above ground material in preparation for winter. Within two hours and upon arrival in the lab, the plant material was chopped into 2 cm segments and dried for 14 h using a LabConco Freezone 4.5 freeze drying unit (Labconco Corporation, Kansas City, MO, USA), followed by storage at −20 °C. The biomass was flash frozen in liquid nitrogen, and pulverized into a fine powder using a mortar and pestle. Approximately 2.0 g of powdered biomass was added to a 250 mL round bottom flask followed by 100 mL of 95% ethanol. The resultant slurry was sonicated for 1 h and then agitated for 24 h on a stir plate. The biomass was removed by vacuum filtration (Whatman filter paper, 0.45 μm), and solvent removed by rotary evaporation. The dried crude extract was dissolved in 10 mL of ethanol, and the solution was warmed to 40 °C and sonicated to achieve complete dissolution. Addition of 35% aqueous ammonia achieved alkaline solvent conditions (pH ≥ 10). The aqueous alkaline solution was added directly to a supported liquid extraction (SLE) column (Chem Elut, Agilent, Santa Clara, CA, USA) and allowed to adsorb for 10 min, followed by elution of alkaloids with chloroform (3 × 10 mL) using a vacuum manifold set to a pressure of 2 mbar. The chloroform fractions were combined, filtered, and evaporated to dryness. All samples were dissolved in 1 mL ethanol as a mixture of alkaloids.

### 4.3. Alkaloid Quantification

The concentrations of cyclopamine, veratramine, isorubijervine and muldamine in alkaloid extracts were determined using an UltiMate 3000 HPLC (Thermo Scientific, Waltham, MA, USA) equipped with a Corona Veo RS charge aerosol detector (CAD) and MSQ Plus mass spectrometer (MS). HPLC separation of alkaloids was achieved using a Thermo Acclaim 120 C_18_ column (2.1 × 150 mm, 3 µm), and mobile phases consisting of 0.1% formic acid (*v*/*v*) in water (Buffer A) and 0.1% formic acid (*v*/*v*) in acetonitrile (Buffer B) with a flow rate of 0.3 mL/min. A linear gradient method beginning at 95% Buffer A and 5% Buffer B, up to 60% Buffer B over a 25 min run time achieved desired separation of alkaloids from the extracts. Cyclopamine, veratramine, isorubijervine and muldamine standards were used to create a calibration curve at concentrations of 0.1, 0.5, 1.0, 5.0 and 10.0 mM with detection recorded by a CAD with the power function set to pA 1.70. Calibration curves were generated in triplicate for each alkaloid at each of the five alkaloid concentrations. The quantity of these alkaloids were determined from the alkaloid mixtures obtained from the leaf, stem and root extracts in triplicate. 

### 4.4. Alkaloid Identification

In order to identify the steroidal alkaloids in *V. californicum* leaf, stem and root/rhizome extracts, samples were analyzed by HPLC-MS, where the mass spectrometer was an ultra-high resolution Quadrupole Time of Flight (QTOF) instrument (Bruker maXis, Billerica, MA, USA). The electrospray ionization (ESI) source was operated under the following conditions: positive ion mode, 1.2 bar nebulizer pressure, 8 L/min flow of N_2_ drying gas heated to a temperature of 200 °C, 3000 V to −500 V voltage between HV capillary and HV end-plate offset, mass range set from 80 to 800 *m*/*z*, and the quadrupole ion energy at 4.0 eV. Sodium formate was used to calibrate the system in this mass range of 80 to 800 *m*/*z*. HPLC separation was achieved using a XTerra MS C_18_ column, 3.5 μm, 2.1 × 150 mm (Waters, Milford, MA, USA). The flow rate was 250 μL/min. The mobile phases were 5% acetonitrile and 0.1% formic acid in water (Buffer A) and acetonitrile and 0.1% formic acid (Buffer B). The linear gradient method was used to separate analytes starting at 5% Buffer B and increasing to 60% Buffer B over 25 min. A 1 μL sample injection was used. Data were analyzed with the Compass Data Analysis software package (Bruker Corporation).

### 4.5. Cell Culture

Shh-Light II cells (JHU-068) were maintained in Dulbecco’s Modified Eagle Medium (DMEM) (Gibco, Carlsbad, CA, USA) supplemented with 0.4 mg/mL geneticin, 0.15 mg/mL Zeocin™ (Invitrogen, Carlsbad, CA, USA), and 10% bovine calf serum. The cells were grown at 37 °C in an atmosphere of 5% CO_2_ in air and 100% relative humidity. This mouse embryonal NIH 3T3 cell line contains a stably transfected luciferase reporter with eight copies of the consensus Gli binding site [35]. Alkaloid treatment conditions were dissolved in ethanol and added to DMEM media containing 0.5% bovine calf serum.

### 4.6. Biological Assays

Cell density was determined using Trypan Blue (Stemcell Technologies, Vancouver, BC, Canada) and a hemocytometer. Shh-Light II cells were seeded in a 96-well plate with 10,000 cells per well, and grown to complete confluence in the media described above. When cells were confluent, the media was replaced with DMEM supplemented with 0.5% bovine calf serum, and treated with 0.1 ng of N-terminal mouse recombinant Shh (R&D Systems, Minneapolis, MN, USA) dissolved in DMEM, and select alkaloid treatment. In each experiment, the controls and treatment wells contained all vehicles, with a final ethanol concentration of 0.05%. Gli activity in the Shh-Light II cell line was assayed 48 h after treatment with Shh protein and select compounds using the Dual-Luciferase Reporter Assay System (Promega, Madison, WI, USA). The Gli-activity was measured by luminescence emitted from cells using a Synergy H1m Microplate reader (BioTek, Winooski, VT, USA). The Gli-activity determined in the biological assay is presented as a relative response ratio (RRR) as described in the Dual-Luciferase Reporter Assay System manual. Each experiment was performed three times.

## Figures and Tables

**Table 1:** Summary of the data corresponding to peaks identified in Figure 1a–c. N/A is used to designate alkaloids with identity not available; N/A^1^ may be etioline or another isomer of isorubijervine; N/A^2^ may be an isomer of veratramine, and N/A^3^ may be an isomer of cyclopamine.

Peak	Retention Time (min)	*m*/*z*	Molecular Formula	Alkaloid
1	12.8	576.3836	C_33_H_53_NO_7_	N/A
2	13.9	572.3530	C_33_H_49_NO_7_	Veratrosine
3	14.6	574.3699	C_33_H_51_NO_7_	Cycloposine
4	14.9	414.3337	C_27_H_43_NO_2_	N/A ^1^
5	15.7	430.3282	C_27_H_43_NO_3_	N/A
6	16.6	428.3136	C_27_H_41_NO_3_	N/A
7	16.7	576.3846	C_33_H_53_NO_7_	N/A
8	16.9	410.3021	C_27_H_39_NO_2_	Veratramine
9	17.4	410.3023	C_27_H_39_NO_2_	N/A ^2^
10	18.7	412.3186	C_27_H_41_NO_2_	Cyclopamine
11	19.5	412.3184	C_27_H_41_NO_2_	N/A ^3^
12	19.7	414.3342	C_27_H_43_NO_2_	Isorubijervine
13	21.1	458.3587	C_29_H_47_NO_3_	Muldamine
14	23.4	400.3550	C_27_H_45_NO	N/A
15	24.5	456.3446	C_29_H_45_NO_3_	N/A

**Table 2:** Quantification of cyclopamine, veratramine, muldamine and isorubijervine by plant part. Alkaloid quantities are reported as mg of alkaloid per g of plant biomass.

Plant Part	Cyclopamine	Veratramine	Muldamine	Isorubijervine
Leaf	0.21 ± 0.02	0.09 ± 0.01	Not Detected	Not Detected
Stem	3.23 ± 0.16	1.33 ± 0.13	0.36 ± 0.06	1.00 ± 0.08
Root/Rhizome	7.38 ± 0.08	3.07 ± 0.14	3.47 ± 0.23	2.92 ± 0.09

## Supplementary Materials

The following are available online; Supplementary S1–S2, Table S1.
