# Supplementary material for: Native V. californicum Alkaloid Combinations Induce Differential Inhibition of Sonic Hedgehog Signaling
Source: Molecules. 2018 Sep 1;23(9):2222. doi: 10.3390/molecules23092222 (PMC6225318; doi:10.3390/molecules23092222)
Supplement: Supplementary file 1 [file molecules-23-02222-s001.pdf]

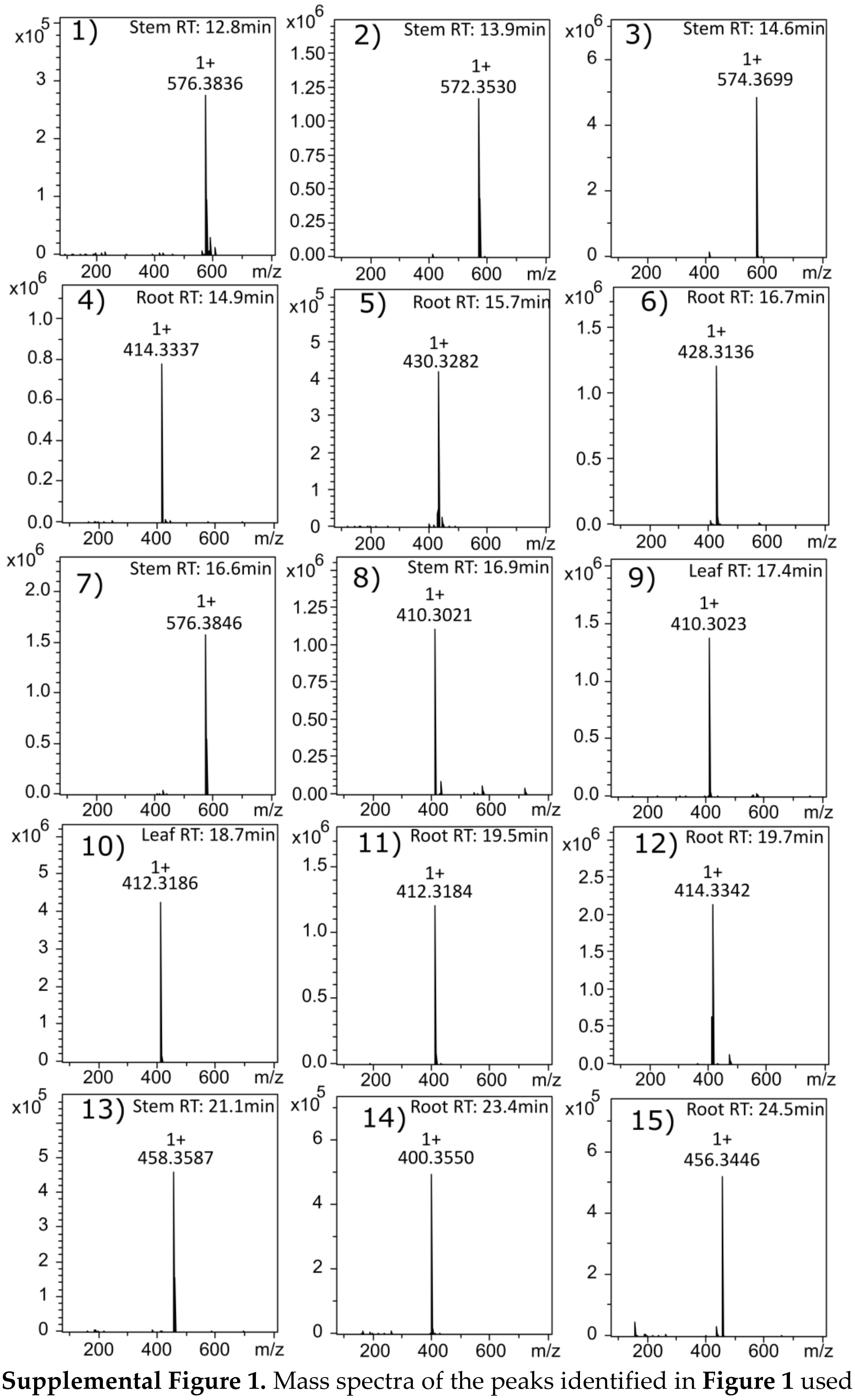

Supplemental Figure 1. Mass spectra of the peaks identified in **Figure 1** used to generate the data in **Table 1**. Included is the retention time (RT) and sample from which the MS data was taken.

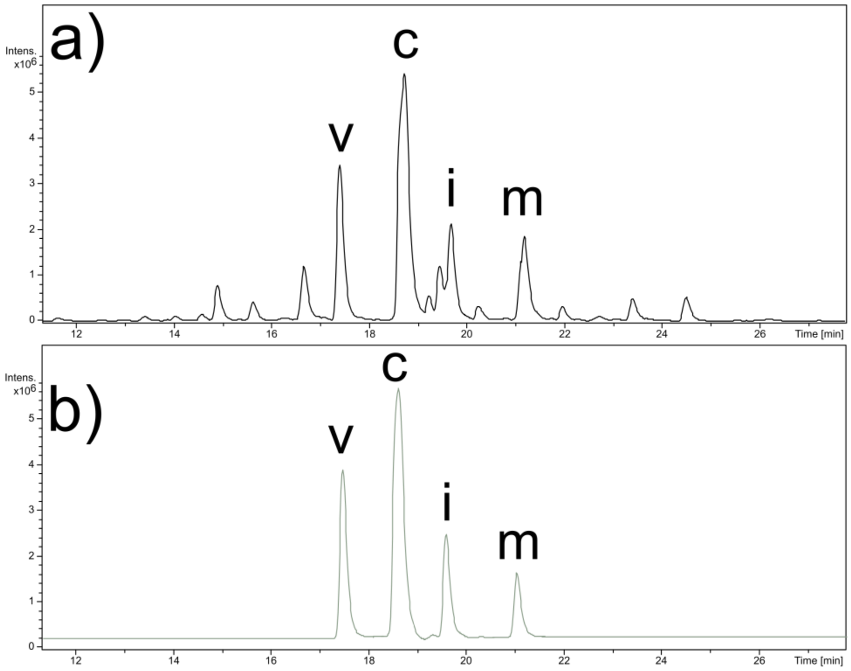

Figure S2. Comparison of **a)** ethanolic root extract and **b)** the root standard mixture, with each commercially available alkaloid indicated as follows: v, veratramine; c, cyclopamine; i, isorubijervine; and m, muldamine. Comparison of the HPLC chromatograms illustrates the quantitative similarity in the concentrations of the alkaloids for which commercial standards are available. Differences in bioactivity between these samples are due to the additional alkaloids present in the extract.

Supplemental Table 2. Bioactivity treatment conditions for Shh-Light II cell assay.

**Treatment Condition**	Cyclopamine (μM)	Veratramine (μM)	Muldamine (μM)	Isorubijervine (μM)
Cyclopamine High	0.5	0	0	0
Leaf Standards High	0.5	0.2	0	0
Stem Standards High	0.5	0.2	0.15	0.05
Root Standards High	0.5	0.2	0.2	0.2
Cyclopamine Low	0.1	0	0	0
Leaf Standards Low	0.1	0.04	0	0
Stem Standards Low	0.1	0.04	0.03	0.01
Root Standards Low	0.1	0.04	0.04	0.04
Leaf Standards High - Cyc	0	0.2	0	0
Stem Standards High - Cyc	0	0.2	0.15	0.05
Root Standards High - Cyc	0	0.2	0.2	0.2
